# *Toxoplasma gondii* Clp family protein: *Tg*ClpB1 plays a crucial role in thermotolerance

**DOI:** 10.18632/oncotarget.20989

**Published:** 2017-09-18

**Authors:** Shinuo Cao, Nali Du, Heming Chen, Yu Pang, Zhaoxia Zhang, Jun Zheng, Honglin Jia

**Affiliations:** ^1^ State Key Laboratory of Veterinary Biotechnology, Harbin Veterinary Research Institute, Chinese Academy of Agricultural Sciences, Harbin, China

**Keywords:** ClpB, caseinolytic proteases and chaperones, AAA+ ATPases, thermotolerance, *toxoplasma gondii*

## Abstract

Caseinolytic peptidase B (ClpB) plays a pivotal role in suppressing and reversing protein aggregation. *Toxoplasma gondii* is an intracellular parasitic protozoan that infects a wide variety of mammals and birds and therefore is exposed to a broad range of living condition. We screened ToxoDB (http://ToxoDB.org) and identified 10 putative *T. gondii* genes encoding members of the Clp superfamily of caseinolytic proteases and chaperones. Of these, we focused on characterizing the Class I ATP-dependent molecular chaperones *Tg*ClpB1, *Tg*ClpB2, and *Tg*ClpB3. We found that *Tg*ClpB1, the most divergent of the five *T. gondii* Class I Clp ATPases, is cytoplasmic, *Tg*ClpB2 is found in the mitochondria of the parasites, and *Tg*ClpB3 is a ClpB with novel apicoplast localization. Knockout strains of *Tg*ClpB1 and *Tg*ClpB2 were established by CRISPR/Cas9 mutagenesis, and their complementing strains were constructed with FLAG-tag. Although knockout of *Tg*ClpB1 or *Tg*ClpB2 did not affect growth under normal circumstances, *Tg*ClpB1 was required for *T. gondii* thermotolerance. The growth, replication, and invasion capabilities of *Tg*ClpB1-deficient mutants were significantly inhibited after extracellular parasites were pretreated at 45°C. Moreover, *Tg*ClpB1 were observed at the poles of the Δ*Tg*ClpB1 FLAG-tagged strain treated at 42°C.

## INTRODUCTION

Caseinolytic proteases and chaperones (Clp) play an important role in protein homeostasis and cellular survival during both stress and optimal growth conditions [1]. The functions of Clp enzymes include the maintenance and repair of salvageable proteins in cells exposed to stress, as well as, disaggregation, translocation, unfolding, and degradation of abnormal or misfolded proteins. Clp chaperone-protease complexes, such as ClpAP, ClpPX, and ClpQY (HslVU), are multimeric ATP-dependent protease systems that are ubiquitous in both prokaryotes and eukaryotes. [2, 3]. These complexes comprise an ATPase and substrate-binding subunit (ClpA, ClpX, ClpY) joined with a proteolytic subunit (ClpP, ClpQ) [4, 5]. An adaptor protein may also be associated, such as ClpS, which interacts with the N-terminal domain of chaperone ClpA and influences the ClpAP complex [6].

*T. gondii* is a unicellular intracellular protozoan belonging to the parasitic phylum Apicomplexa and subclass Coccidium [7]. It is one of the most widespread parasites in the worldwide, potentially capable of infecting all birds and mammals including humans [8]. The parasite must adapt to a board range of temperatures during its complex life cycle, existing in hosts such as cattle (36.7-39.1°C), cats (38.6-40.1°C), dogs (37.9-39.9°C), chickens (39.6-43.6°C), and geese (40.0-44.0°C). Furthermore, the parasitic infection might induce a systemic inflammatory reaction that can elevate the temperature of the host. High temperature stress has a wide range of effects on cells, including changes in membrane fluidity and structure, cell cycle arrest, protein denaturation, and cell death. Such issues can occur when essential proteins are damaged by high temperatures, or when aggregates of damaged proteins become toxic to the cell [9, 10]. Clp superfamily proteins are crucial for protein quality control under these conditions, and yet little is known about this family of proteins in *T. gondii*.

ClpB and its orthologs (ClpA, ClpC, ClpD, ClpX, and ClpY), also known as heat shock protein 100 (HSP100)/Clp, are members of the ATPases Associated with diverse cellular Activities (AAA) superfamily, and is able to recover aggregated proteins in an ATP-dependent manner [11]. Notably, ClpB family members are specific to bacteria, cyanobacteria, yeast, plants, and parasitic protozoans and are not present in animals or humans [12]. Yeast ClpB and its bacterial homologs can disaggregate insoluble proteins caused by severe stresses [13]. *Plasmodium falciparum* ClpB1 (*Pf*ClpB1) is located at the parasite’s apicoplast and is involved in metabolic pathways necessary for the organism’s survival [14–16]. *Pf*ClpB2 is targeted to the parasitophorous vacuole membrane (PVM), where it acts as an essential subunit of the *Plasmodium* translocon of exported proteins (PTEX) complex [17]. In *Leishmania major*, the ClpB proved essential for the growth and survival of parasites infecting mouse macrophages *in vitro* [18, 19]. We sought to determine whether ClpB family members identified by searching the *T. gondii* genome, might also be involved in maintaining parasitic proteostasis. In this study, we present an identification and functional analysis of *T. gondii*’s Clp superfamily of proteins. Our results demonstrate that *Tg*ClpB1, *Tg*ClpB2 and *Tg*ClpB3 localize in the cytosol, mitochondria and apicoplast of *T. gondii*, respectively. Additionally, we found that *Tg*ClpB1 plays an essential role when cells are exposed to extreme heat.

## RESULTS

### The Clp superfamily proteins in *T. gondii*

**Figure 1:**
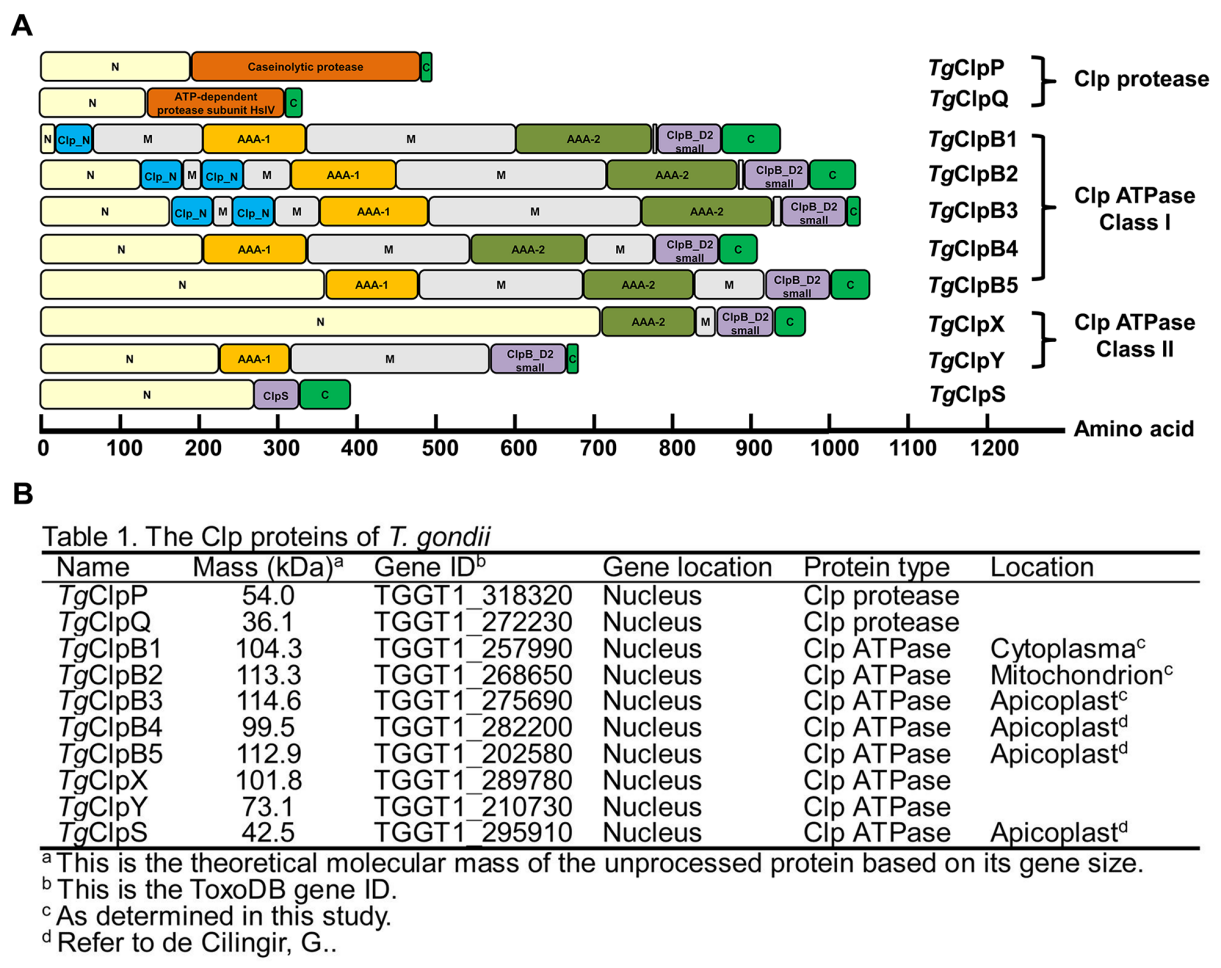
**(A)** Comparison of the conserved domains of the Toxoplasma gondii Clp proteases and ATPases. The ClpB1 protein of Class I can be identified by their two AAA domains, whereas Class II proteins contain a single AAA domain. The AAAs (AAA-1, orange; AAA-2, olive) are highly conserved, whereas the N domains (yellow), M domains (gray), and C domains (green) have low sequence homology. Other identified domains include ClpB_D2 depicted in purple, Clp_N in light blue, and the Clp protease in dark orange. **(B)** Table 1. Bioinformatics analysis of the Clp superfamily proteins in *T. gondii*.

Ten putative clp genes were identified in *T. gondii* (Figure 1). All sequences were found on the nuclear chromosomes. Two *T. gondii* sequences (TGGT1_318320, *Tg*ClpP and *TG*GT1_272230, *Tg*ClpQ) were found to have a high level of sequence homology with *P. falciparum* 3D7 ATP-dependent CLP protease (*Pf*ClpP, PF3D7_0307400) and ATP-dependent protease subunit ClpQ (*Pf*ClpQ, PF3D7_1230400), respectively. *Tg*ClpP was identified as caseinolytic protease by its S14_ClpP_2 domain (residues 193 to 477), containing conserved active site residues and an oligomer interface. We found that *Tg*ClpQ had a PRK05456 domain (residues 136 to 306), and therefore classified the protein as a homolog of the ATP-dependent protease subunit HslV.

*T. gondii* Clp Class I include *Tg*ClpB1, *Tg*ClpB2, *Tg*ClpB3, *Tg*ClpB4 and *Tg*ClpB5. All of these proteins are predicted to contain two ATP nucleotide-binding domains (AAA domains) that have characteristic Walker A and Walker B nucleotide-binding and -recognition motifs. As DNASTAR program protean prediction, the five *Tg*ClpBs present the long coiled-coil domains inserted between the two AAA domains, which is a characteristic of ClpB. The parasite contains two members of Clp ATPases Class II, which we named *Tg*ClpX and *Tg*ClpY. ClpY is the ATPase and the substrate-binding subunit. The ClpY molecule is distinguished by two domains: the AAA domain (residues 225 to 312). ClpS shares a very high degree of homology with *P. falciparum* ClpS (*PF*3D7_1320100). Of the family members we have identified, only *Tg*ClpS, *Tg*ClpB4, and *Tg*ClpB5 contain predicted apicoplast-targeting sequences [20]. SignalP 4.1 server (http://www.cbs.dtu.dk/services/SignalP/) predicted the absence of signal peptide cleavage sites in amino acid sequences of these TgClp family proteins.

### Generation of *Tg*ClpB1, *Tg*ClpB2 and *Tg*ClpB3 knockout and complementing strains

**Figure 2:**
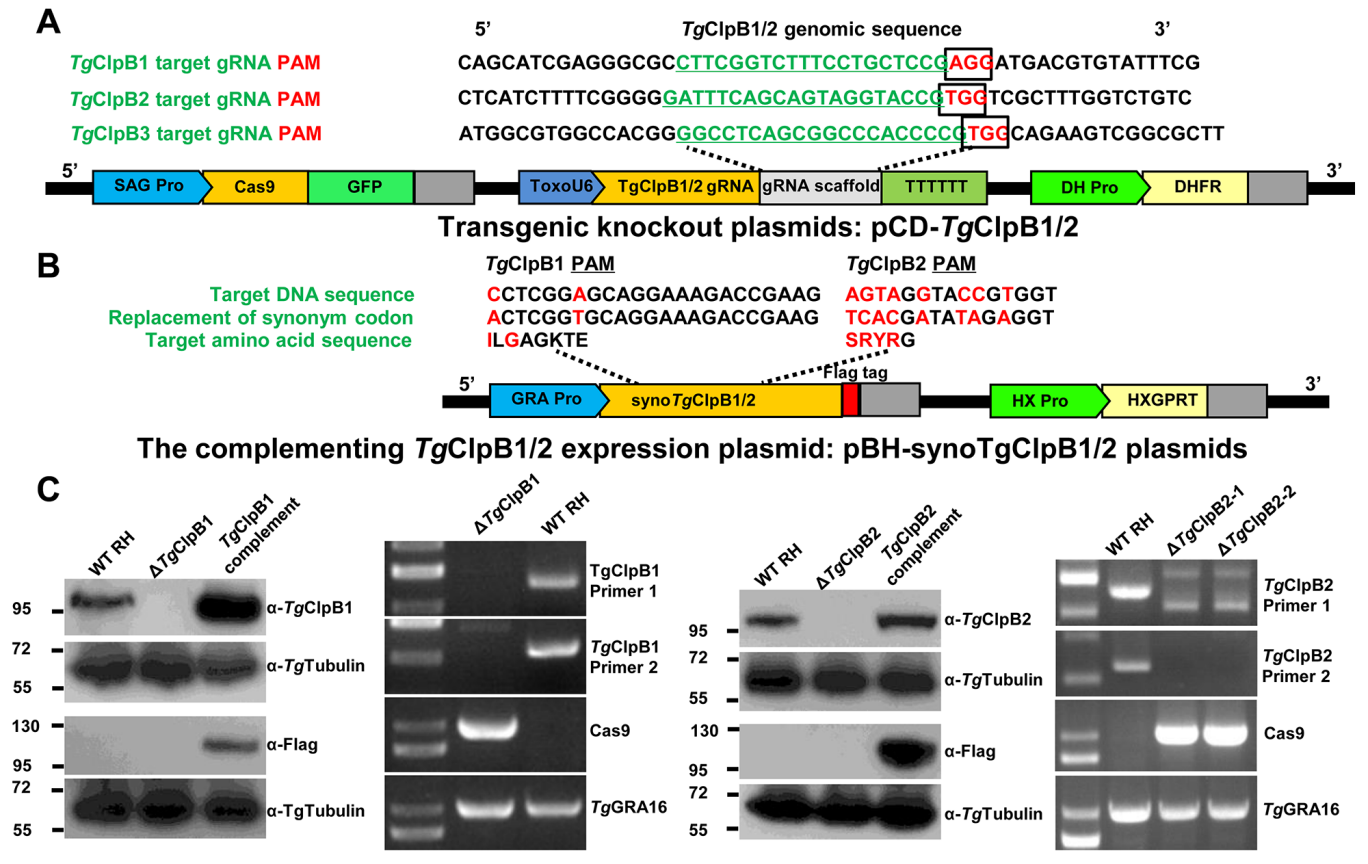
**(A)** The TgClpB1 and TgClpB2 disruption sites are shown in their genomic context. The target guide RNAs (gRNA) of *Tg*ClpB1 and *Tg*ClpB2 are underlined, and the protospacer adjacent motif (PAM) for each is framed. The pCD-*Tg*ClpB1 and pCD-*Tg*ClpB2 expression plasmids contain three important cassettes in addition to the *Tg*ClpB1, *Tg*ClpB2 or *Tg*ClpB3 gRNA: GFP, Cas9, and dihydrofolate reductase (DHFR). **(B)**, **(C)** PCR and Western blot analysis confirmed the respective absence of *Tg*ClpB1 and *Tg*ClpB2 in the Δ*Tg*ClpB1 andΔ*Tg*ClpB2 strains as well as the presence of the proteins in the parental HX-RH, Δ*Tg*ClpB1::*Tg*ClpB1-FLAG and Δ*Tg*ClpB2::*Tg*ClpB2-FLAG tachyzoites.

To investigate the function of *Tg*ClpB1, *Tg*ClpB2, and *Tg*ClpB3 in *T. gondii*, we proceeded to generate the knockout mutants (Δ*Tg*ClpB1, Δ*Tg*ClpB2 and Δ*Tg*ClpB3). The gene-targeting plasmids, designated pCD-*Tg*ClpB1, pCD-*Tg*ClpB2 and pCD-*Tg*ClpB3 (Figure 2A), were constructed using specific gRNAs with a DHFR selectable marker cassette. Plasmid pBluescript II containing codons synonymous to *Tg*ClpB1, *Tg*ClpB2 and *Tg*ClpB3 was then fused with FLAG-tag and a hypoxanthine xanthine guanine phosphoribosyl transferase (HXGPRT) expression cassette, designated pBS-syno-*Tg*ClpB1, pBS-syno-*Tg*ClpB2 and pBS-syno-*Tg*ClpB3 (Figure 2B). After electroporation and selection, Δ*Tg*ClpB1 and Δ*Tg*ClpB2, their complement, and the Cas9 control strains were all verified by Western blot analysis (Figure 2). Protein bands of approximately 100 kDa were observed in the lysate of the Cas9 control cell line, but not in the Δ*Tg*ClpB1 or Δ*Tg*ClpB2 strains. This size correspond to the predicted size of the proteins (104311.39 Da and 114596.39 Da) listed in ToxoDB. The 100 kDa band was also observed after probing the complementing strain lysates with anti-*Tg*ClpB1 or *Tg*ClpB2 serum, as appropriate. The presence of the proteins was additionally confirmed using anti-FLAG monoclonal antibody. To further verify *Tg*ClpB1 and *Tg*ClpB2 knockout, the mutant strains were also examined by PCR assay (Figure 2). Together, these experiments confirmed the respective absence of *Tg*ClpB1 and *Tg*ClpB2 in the Δ*Tg*ClpB1 and Δ*Tg*ClpB2 strains. We also attempted to generate the *Tg*ClpB3 knockout strain by introducing the pCD-*Tg*ClpB3 plasmid in three independent experiments. However, the transfected parasites failed to grow after addition of pyrimethamine. Our findings suggest that *Tg*ClpB3 may be crucial for the parasites survival.

### *Tg*ClpB1 and *Tg*ClpB2 are not required for trafficking of *Tg*GRA16 and *Tg*GRA24 to the host cell

*Tg*ClpB1 and *Tg*ClpB2 were found to have a high level of sequence homology with *P. falciparum* ClpB2/HSP101 (*PF*11_0175), which is an essential protein for export of diverse malaria effectors into host erythrocytes (Supplementary Figure 1). We suspected that *Tg*ClpB1 and *Tg*ClpB2 may have a similar function in assisting the transport of *Tg*GRA16 and *Tg*GRA24 from the PVM to the host nucleus. We therefore examined the location of *Tg*GRA16 and *Tg*GRA24 in the Δ*Tg*ClpB1/2 mutants by immunofluorescence in infected cells. To do this, we introduced a plasmid expressing either HA-tagged *Tg*GRA16 or HA-tagged *Tg*GRA24 into the RH-WT and Δ*Tg*ClpB1/2 strains. Infected cells were stained at 24 hpi, and this revealed that *Tg*GRA16 and *Tg*GRA24 from both knockout strains were still able to locatize to the host cell’s nucleus. This indicates that *Tg*ClpB1 and *Tg*ClpB2 are not required for exporting proteins from *T. gondii* to host cells (Supplementary Figure 2).

### *Tg*ClpB1 is cytoplasmic and redundant under optimal conditions

**Figure 3:**
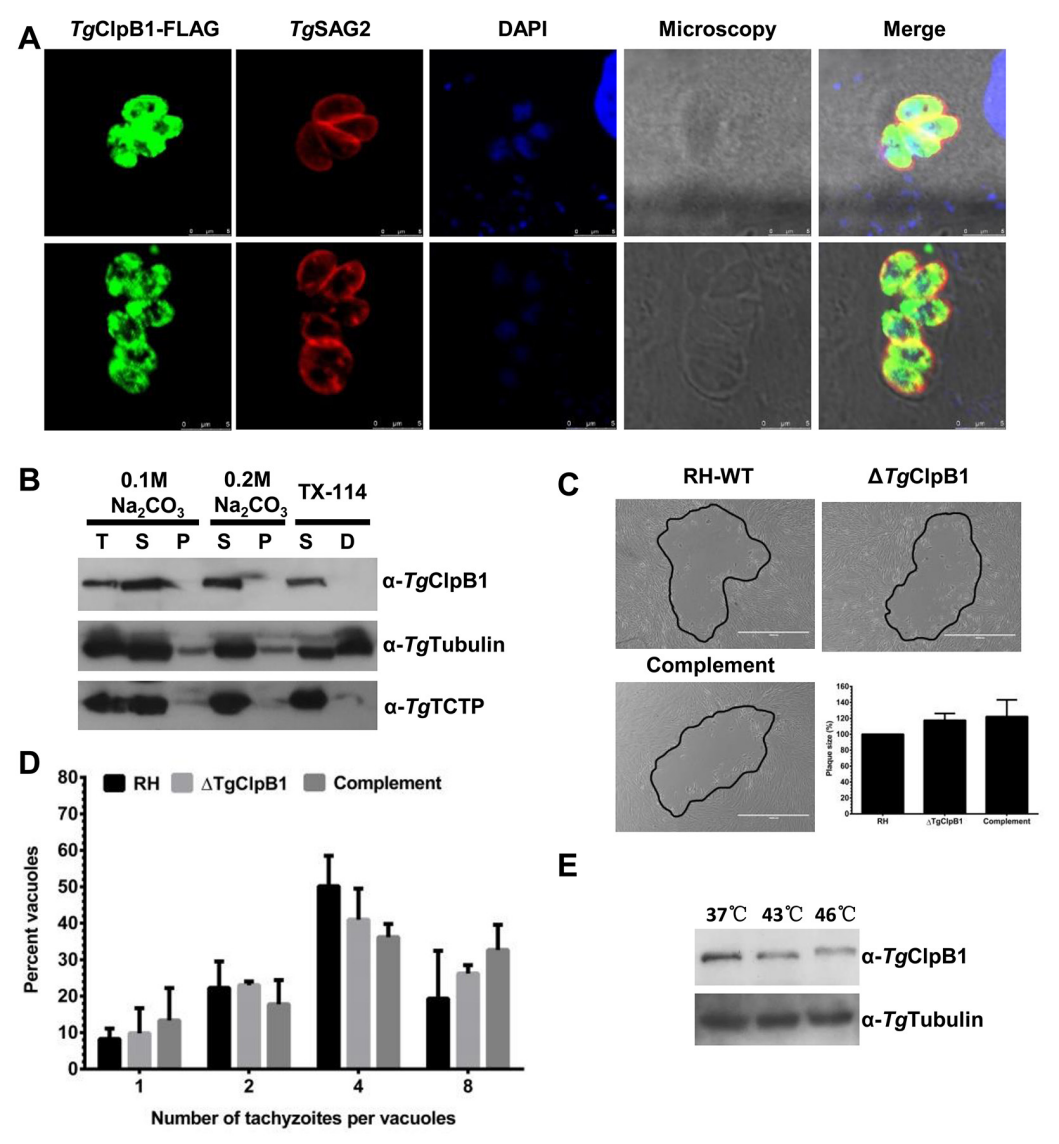
**(A)** TgClpB1-FLAG colocalizes with the cytosolic marker TgSAG2. **(B)** TgClpB1 is a soluble protein. Cytosolic proteins were extracted from RH-WT parasites and separated by either sodium carbonate treatment into soluble (S) and membrane pellet (P) fractions or by Triton X-114 phase partitioning into soluble (S) and detergent (D) fractions. The first lane displays total protein extract (T). **(C)** Wild-type, knockout, and complemented strains were grown in HFF cells for 7 days. A representative plaque is shown for each, outlined in black. **(D)** Intracellular replication was not changed at 37°C for the ΔTgClpB1 strain. **(E)** TgClpB1 was detected by Western blot in extracellular parasites treated at 37°C, 43°C, or 46°C for 4 h.

Unlike *Pf*ClpB2, *Tg*ClpB1 was not detected in the PVM of *T. gondii*. *Tg*ClpB1-FLAG instead presented as a soluble protein in the parasitic cytoplasm of *T. gondii* (Figure 3A and 3B). Growth of the Δ*Tg*ClpB1 mutant as measured by plaque formation and intracellular replication was normal when the extracellular parasites were maintained under the standard conditions of 37°C, 30 min (Figure 3C and 3D). The egress rate of Δ*Tg*ClpB1 was also not significantly different compared with RH-WT and *Tg*ClpB1 complement strain at optimal condition (data not shown). To explore the knockout parasite’s response to heat stress, we next characterized the expression levels of *Tg*ClpB1 after heat treatment at 43°C or 46°C for 4 h. We observed that the protein band corresponding to *Tg*ClpB1 shifted to a higher molecular weight at 46°C, but not at 43°C (Figure 3B).

### *Tg*ClpB1 is necessary for thermotolerant growth and replication of *T. gondii* tachyzoites

**Figure 4:**
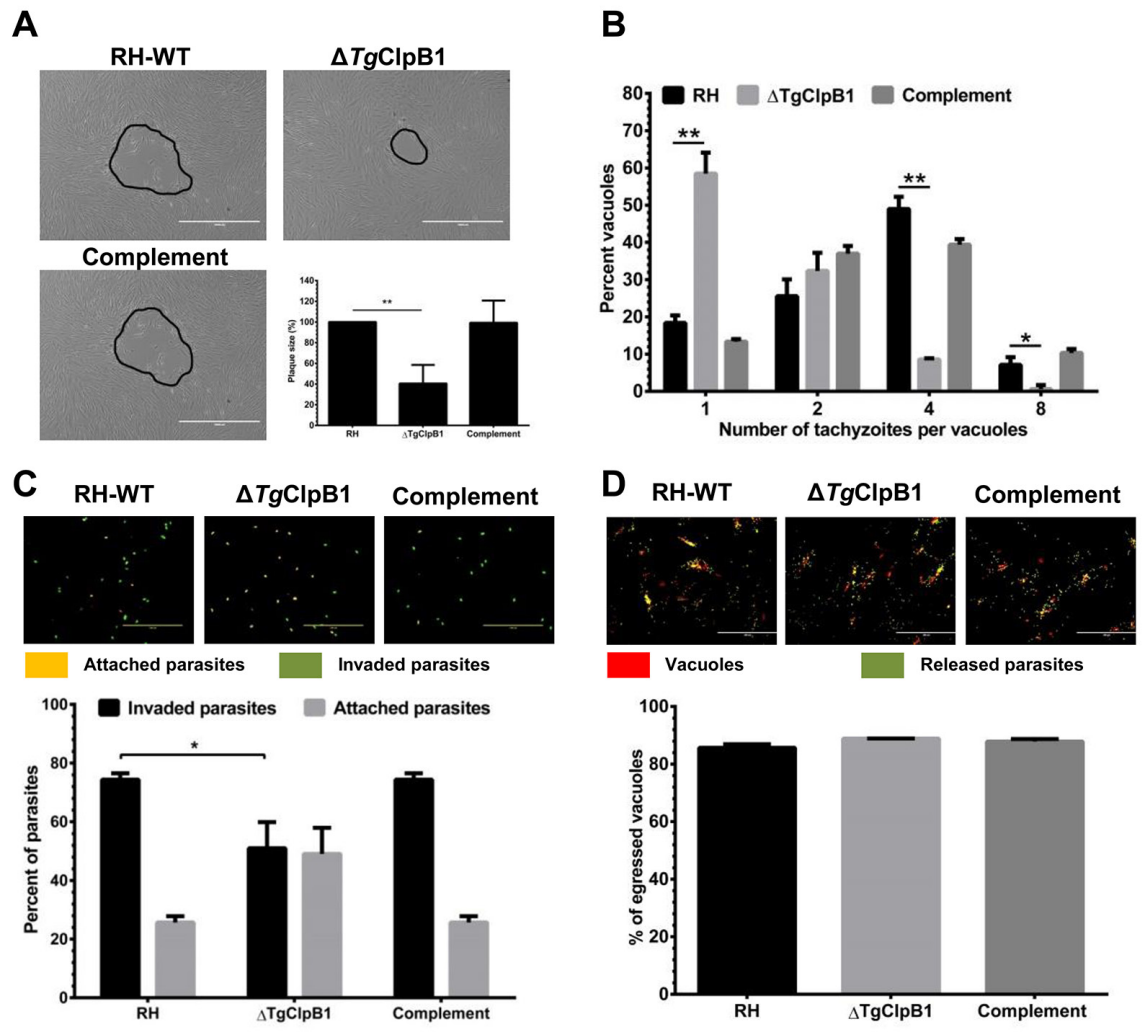
**(A)** When parasites were grown in HFF cells after the released parasites treated at 45°C, ΔTgClpB1 led to a reduction in plaque formation compared with RH-WT. Representative plaques are shown outlined in black. **(B)** The ΔTgClpB1 strain displays decreased intracellular replication at the same condiction. Parasites were allowed to invade Vero cells for 3 h. After 24 h, the parasites were fixed and stained with 4% paraformaldehyde. The percentage of vacuoles containing one, two, four, or eight parasites was determined. Results are shown as an average of three independent experiments. **(C)** The invasion efficiency of the ΔTgClpB1 strain was significantly reduced when the extracellular parasites were treated at 45°C. Below, mean values of three independent experiments are graphed with bars representing standard deviation. **(D)** ΔTgClpB1 mutant does not affect in ability to egress from host cells following stimulation of calcium signaling with calcium ionophore. A23187.

Aiming to further understand how *Tg*ClpB1 influences parasite viability, we examined the growth and replication capacities of the Δ*Tg*ClpB1 strain under heat shock conditions. The gradient temperature actions were performed to treat freely egressed RH-WT, Δ*Tg*ClpB1 and complement parasites at 37, 39, 42, 45, 47 or 50°C. The results showed that the growth of the parasites was universally inhibited at 47°C. However, apparent differences were able to be observed between RH-WT and Δ*Tg*ClpB1 strains at 45°C (Supplementary Figure 3). Thereafter, compared with its controls, growth of the Δ*Tg*ClpB1 strain was severely impaired after treatment at 45°C for 30 min. We observed a marked reduction in plaque formation consistent with the decreased intracellular replication of the Δ*Tg*ClpB1 mutant. This further supported the crucial role of Δ*Tg*ClpB1 in the growth and replication of *T. gondii* tachyzoites under heat stress conditions (Figure 4B).

### Loss of *Tg*ClpB1 reduced the invasion rate of the parasites after the heat stress

To investigate whether the *Tg*ClpB1 knockout strain had any defect in infectivity at 45°C, we performed an attachment/invasion assay. Evaluating the cellular invasion rate of the knockout parasites showed a significant difference in attachment/invasion behavior between RH-WT and Δ*Tg*ClpB1 strains (*P* < 0.05) (Figure 4C). These results indicate that the loss of Δ*Tg*ClpB1 reduces the parasite’s invasion ability under heat stress *in vitro*. Furthermore, the results reveal that Δ*Tg*ClpB1 did not affect the egress capacity of the parasites after the heat stress treatment (Figure 4D).

### Heat shock induces polar aggregation within the parasites

**Figure 5:**
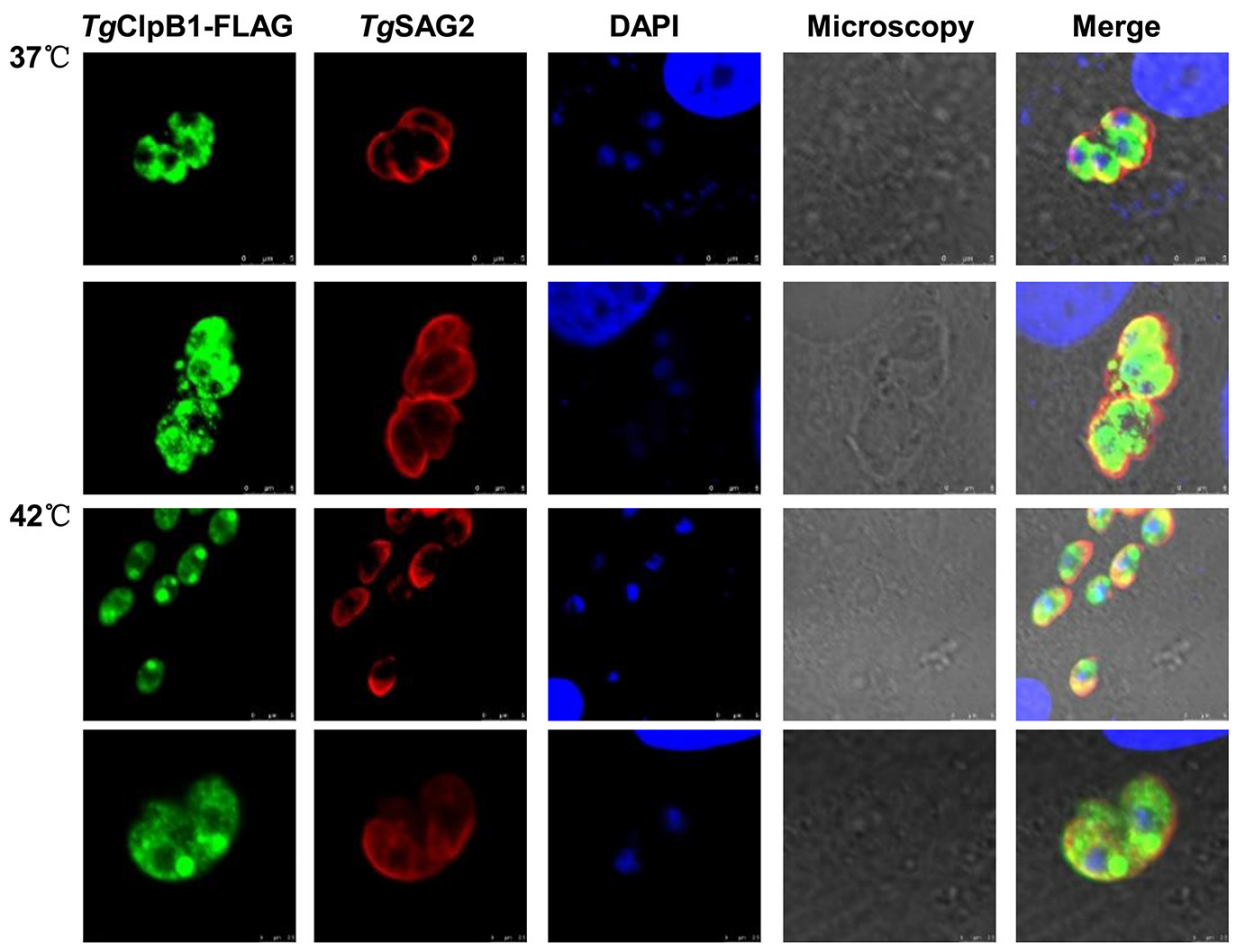
TgClpB1-FLAG aggregates at the parasitic poles under heat stress conditions Vero cells were infected with parasites expressing Flag-tagged *Tg*ClpB1. After treatment at 42°, the cultures were washed, fixed, and stained with antibodies to the Flag tag and *Tg*SAG2.

To investigate whether *Tg*ClpB1 is activated upon heat shock, the *Tg*ClpB1-FLAG was detected by immunofluorescence in intracellular parasites. When incubated at 42°C, the *Tg*ClpB1-FLAG proteins display an increased localization at the poles of the parasites when compared with the diffuse cytosolic staining observed at 37°C. In individual parasites, one or two inclusion bodies were observed (Figure 5).

### *Tg*ClpB2 has mitochondrial localization and is not required for thermotolerance

**Figure 6:**
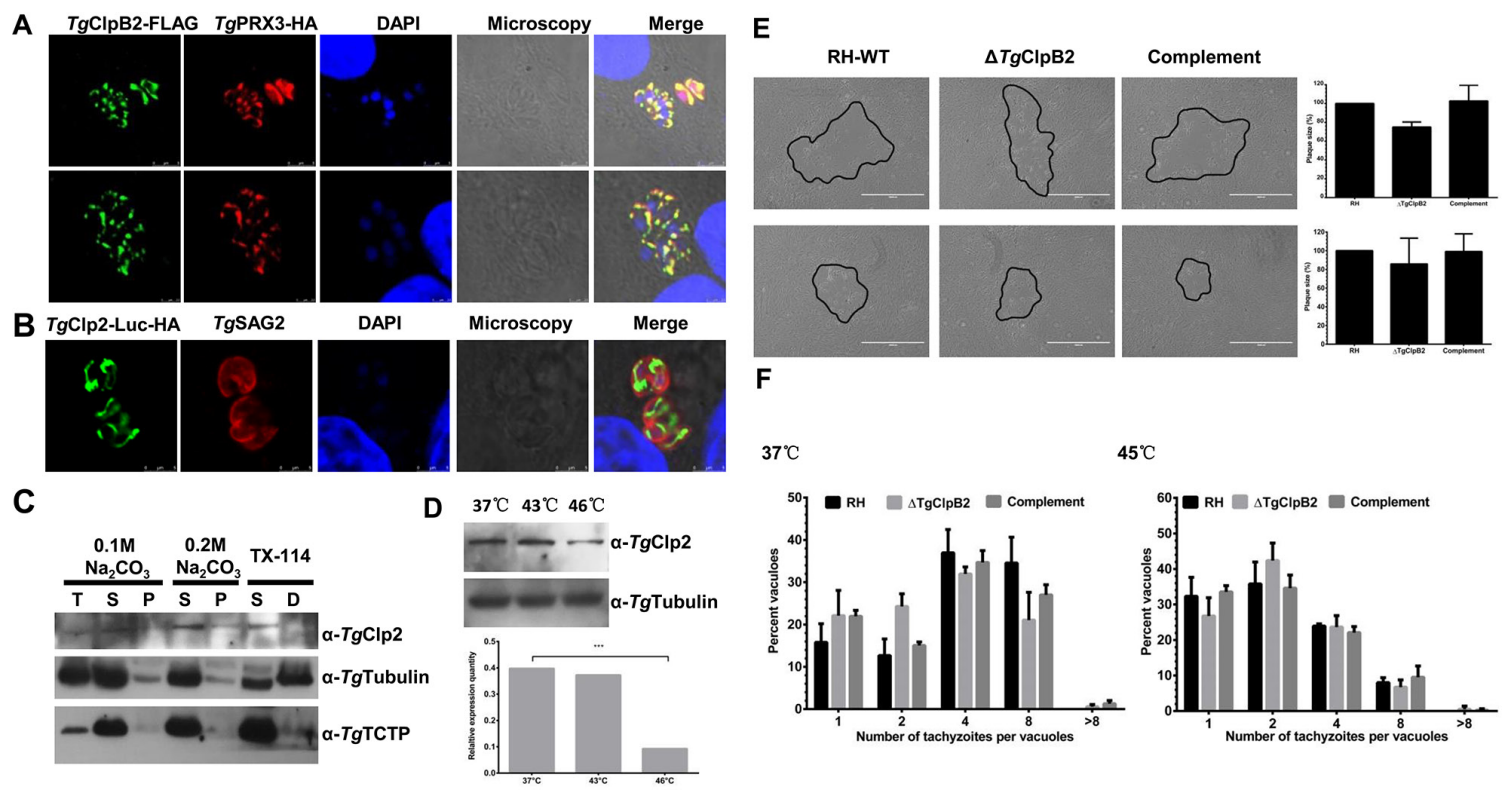
**(A)** Colocalization of TgClpB2-FLAG with the mitochondrial marker TgPRX3-HA suggests that TgClpB2 is targeted to the mitochondrion. **(B)** When fused to the reporter protein Luc-HA, the predicted transit peptide *Tg*ClpB2 (1-113) is sufficient to induce mitochondrial localization of the construct. **(C)** *Tg*ClpB2 is present in both the soluble and insoluble fractions of the parasitic extract. **(D)** *Tg*ClpB2 is detectable by Western blot in extracellular parasites treated at 37°C, 43°C, or 46°C for 4 h. **(E)** *T. gondii* thermotolerance is independent of *Tg*ClpB2. When grown in HFF cells for 7 days, no significant alteration of plaque formation is seen with the Δ*Tg*ClpB2 strain as compared with its controls. Representative plaques are outlined in black. **(F)** Loss of *Tg*ClpB2 does not significantly alter intracellular replication rate at either 37°C or 45°C.

The localization of *Tg*ClpB2-FLAG was observed at the mitochondria of parasites with *Tg*PRX3-HA (Figure 6A). To characterize the expression profile of *Tg*ClpB2, the parasite lysates were detected by Western blot analysis using mouse anti-*Tg*ClpB2 antibody. *Tg*ClpB2 was detectable within both the soluble and insoluble fractions (Figure 6C). We also observed that *Tg*ClpB2 expression declined when the parasites were exposed to higher tempretures (Figure 6D). However, when the growth and replication rates of Δ*Tg*ClpB2 strains were examined under optimal and heat shock conditions, no significant change was observed as compared with the RH-WT and complementing strains.

### N terminal signal of *Tg*ClpB2 targets the mitochondria of parasites

To test whether *Tg*ClpB2 contains a targeting sequence for the *T. gondii* mitochondria, Luc fusion protein constructs were made using the *Tg*ClpB2 pre-sequence (the first 1113 amino acids). This gene fusion construct successfully yielded a pattern of mitochondrial fluorescence when introduced to the parasite. Therefore, the first 113 amino acids of the *Tg*ClpB2 sequence appear to be sufficient to target proteins to the mitochondria of *T. gondii* (Figure 6B).

### *Tg*ClpB3 is a membrane associated protein located at the apicoplast of the parasites

**Figure 7:**
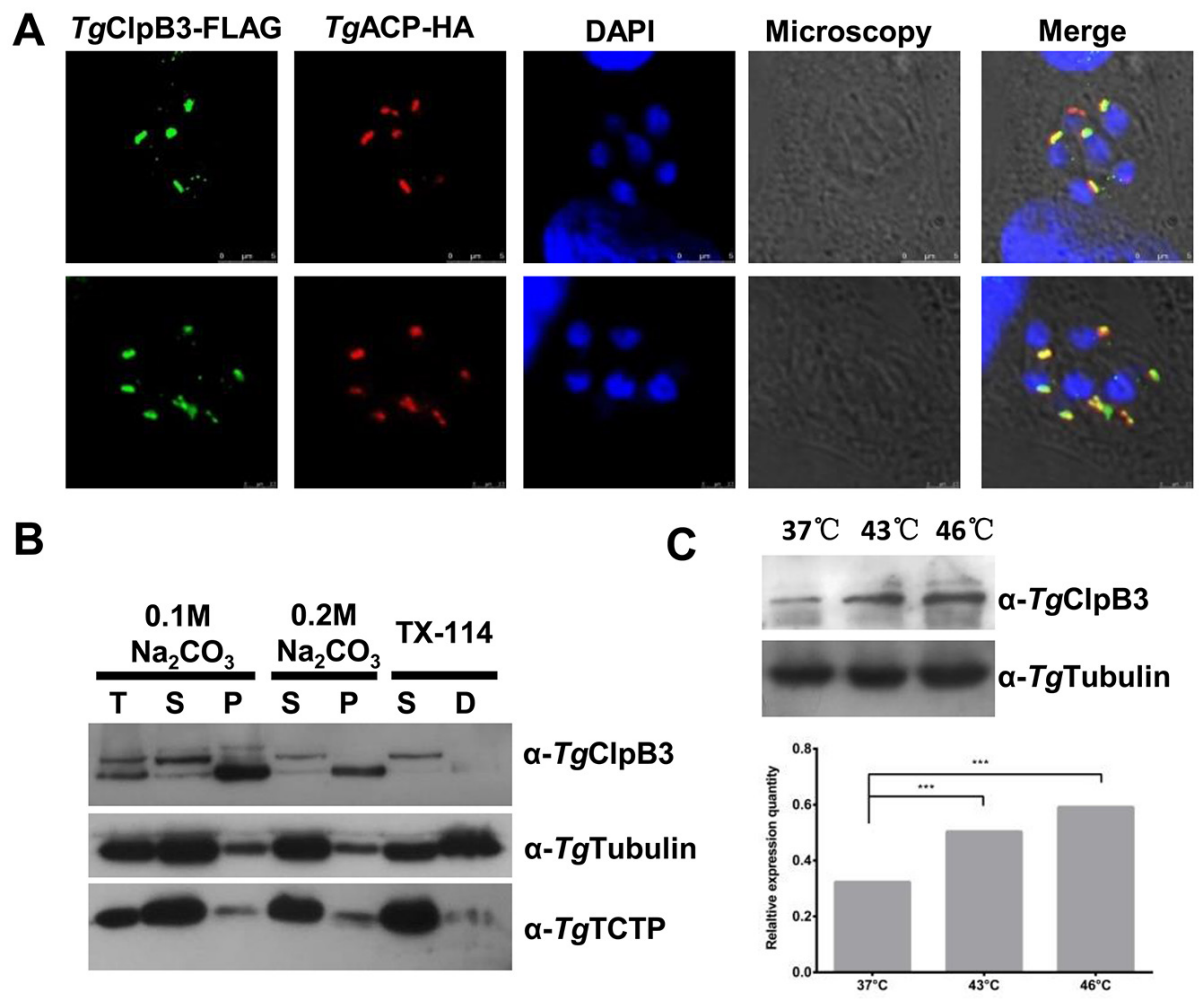
**(A)** Colocalization of TgClpB3-FLAG with the marker TgACP-HA suggests that TgClpB3 is targeted to the apicoplast of *T. gondii*. **(B)** *Tg*ClpB3 is a membrane-associated protein. Proteins were extracted from RH-WT and either separated into soluble (S) and membrane pellet (P) fractions by sodium carbonate treatment or into soluble (S) and detergent (D) phases by Triton X-114 phase partitioning. Total protein extract (T) is shown in the first lane. **(C)** Western blot detection of *Tg*ClpB3 in extracellular parasites treated at 37°C, 43°C, or 46°C for 4 h.

To examine the location of *Tg*ClpB3, HFF monolayers were infected with a parasitic *Tg*ACP-HA overexpression strain. The *Tg*ClpB3 was then detected by mouse anti-*Tg*ClpB3 polyclone antibody. The protein appeared to colocalize with the *Tg*ACP-HA marker at the apicoplast of the parasites (Figure 7A). To evaluate whether *Tg*ClpB3 is membrane associated, we next performed sodium carbonate extraction and Triton X-114 phase partitioning. Western blot analyses revealed that *Tg*ClpB3 was present in both unprocessed and mature forms in the total parasite lysate. The unprocessed version was mainly exhibited at the soluble fraction (Figure 7B), whereas the mature form was mainly detected at the insoluble and membrane fractions. Furthermore, *Tg*ClpB3 appeared to be strongly up-regulated after heat treatment at 43°C or 46°C for 4 h (Figure 7C).

## DISCUSSION

Here, we have provided the first integrated survey of the Clp chaperones and proteases in *T. gondii*. We found that the parasite contains two proteolytic subunits, which we term *Tg*ClpP and *Tg*ClpQ. Five Class I Clp chaperones (*Tg*ClpB1, *Tg*ClpB2, *Tg*ClpB3, *Tg*ClpB4 and *Tg*ClpB5), and two Class II Clp chaperones (*Tg*ClpX and *Tg*ClpY). *Tg*ClpS, which is responsible for the specific recognition of proteins bearing an N-terminal destabilizing residue, was also identified. In *P. falciparum*, *Pf*ClpQ directly interact with the C-terminus of *Pf*ClpY to form a protein complex in mitochondria, which is crucial for the growth and survival of asexual stage parasites [21, 22]. Generally, ClpXP is known to be an ATP-powered protease that can unfold and degrade aggregated proteins, performing essential functions of protein quality control as it participates in numerous regulatory circuits from bacteria to humans [23]. Although the *T. gondii* ClpP, ClpQ, ClpX and ClpY proteins were not characterized in this study, the parasite’s genome harbors two potential Clp protease systems: ClpXP and ClpQY. These prokaryotic-specific ATP-dependent protease complexes may represent potential drug targets in *T. gondii*.

ClpB and its orthologs involved in cellular quality control belong to the AAA superfamily. These enzymes are typified by an N-terminal domain and two ATP-binding domains (AAA domains) essential for hexamerization and chaperone function [12]. *T. gondii* contain five ClpB proteins. In *Leishmania*, ClpB is membrane-associated and crucial for the secretion of exosomes [19]. For *P. falciparum*, ClpB2 is a major component of PTEX and is required to mediate protein export to parasite’s host cell [17]. Although *Tg*ClpB1 and *Tg*ClpB2 both have a high level of sequence homology with *Pf*ClpB2 (*PF*11_0175), *Tg*ClpB1 and *Tg*ClpB2 were localized at the cytoplasm and mitochondria of parasite, respectively. Neither of the knockout strains limited the export of *Tg*GRA16 and *Tg*GRA24 into the nucleus of the host cells. The *T. gondii* homologues of *Pf*EXP2 are *Tg*GRA17 and *Tg*GRA23, which located at the PVM and are responsible for the transfer of small molecules though the PVM [24]. In a recent report, MYR1 is suggested to be a component of machinery that media the transportation of GRA proteins into the cytosol of host cells [25]. Bath on our results, it is possible that *T. gondii* utilizes a complete different pathway for the transportation of the proteins from the PVM.

The formation of misfolded proteins is an inevitable process in living cells, which can cause cellular toxicity if left unchecked. Misfolding can be enhanced by internal and environmental stresses such as heat shock [26, 27]. ClpB acts to counter protein aggregation in the cytosol of bacteria, yeast, and plants, and thereby ensures the proper functioning of proteins in their cellular environment [28–31]. Through FLAG-tag constructs, we determined that the subcellular localization of *Tg*ClpB1 and *Tg*ClpB2 is, respectively, within the cytoplasm and mitochondria of the parasites. Moreover, the shifted molecular weight was also observed at the *Tg*ClpB1 after heat treatment. In *Chlamydomonas*, the molecular weight of HSF1 protein is observed to increase slightly between 15 and 30 min after the onset of heat shock, a change that has been demonstrated to be due to phosphorylation [32]. The higher molecular weight may be due to modifications of the activated protein after heat shock. As in the previous study, the higher molecular weight of *Tg*ClpB1 may be due to an activating modification that occurs as a result of heat shock. Furthermore, we discovered that disruption of *Tg*ClpB1 results in a severe defect in *T. gondii* invasion and intracellular growth after following heat shock. The specific action of ClpB1 in *T. gondii* may be similar to that of its homologous cytoplasmic protein in yeast, since both are directly involved in the organism’s thermotolerance.

We additionally determined that *Tg*ClpB1 is soluble, not a membrane-associated protein. Immunofluorescence analysis revealed that *Tg*ClpB1 migrates and displays polar localization after heat shock. Although the 42°C treatment used for our experiments has no growth effect on wild-type *T. gondii*, it does induce observable, polar-localized protein aggregations in the parasites. To our knowledge, this is the first observation of these inclusion bodies in *T. gondii*. In other organisms, previous reports in bacteria have detailed misfolded proteins observed to aggregate in inclusion bodies given various conditions. Moreover, yeast cells also accumulate aggregated, insoluble proteins at the perivacuolar insoluble protein deposit (IPOD) [31]. In mammals, an aggresome is assembled with misfolded and ubiquitylated proteins at an indentation of the nucleus surrounding the centriole [33]. Taken together, our results suggest that *T. gondii* may have a protein quality control mechanism similar to the yeast and bacterial response to protein aggregation.

*Tg*ClpB2 were detected at the mitochondria of the parasites by tagging the respective parasite genes with FLAG tag. To test the putative transit peptide of *Tg*ClpB2 present on nuclear-encoded mitochondrion proteins mediate targeting, Luc fusion proteins were constructed by using the putative N-terminal targeting transit peptides. The results demonstrated that the 113-aa N-terminal presequence of *Tg*ClpB2 is sufficient to target a protein into the mitochondrion. The declined expression of *Tg*ClpB2 after heat treatment was observed in this study. In previous study, the decreased expression of heat shock protein 70 mRNA and protein were also observed after heat treatment in cells of aged rats although the mechanism is still unclear [34]. On the other hand, the Mitochondrial ClpB Homolog Hsp78 of the *Saccharomyces cerevisiae* is indispensable for the resolubilization of protein aggregates generated by heat stress under *in vivo* conditions. However, our results reveal that the disruption of *Tg*ClpB2 does not affect the growth and replication capacities of *T. gondii* under optimal and heat stress conditions. The function of *Tg*ClpB2 needs to be characterized in the further study.

Finally, we have been able to determine that the *Tg*ClpB3 is located at the apicoplast of the parasites. A previous bioinformatic study implied that *Tg*ClpB4 and *Tg*ClpB5 are located in the apicoplast of parasites using a parametric model for ApicoTPs [20]. We found by Western blot analysis that the unprocessed and mature forms of *Tg*ClpB3 are separately in soluble and insoluble fraction, respectively. Although the *Tg*ClpB3 does not have transmembrane domain, in the present study the mature form appeared to be sequestered in the insoluble and membrane-associated fractions. This suggests that *Tg*ClpB3 may be a part of an insoluble complex on the membrane of the parasitic apicoplast. The apicoplasts of Apicomplexa parasites are organelles that originated from an alga through secondary endosymbiosis. They contain several essential metabolic pathways such as heme, isoprenoid, and fatty acid biosynthesis. A resent study demonstrated that TIC/TOC machinery is involved in the import mechanisms across the innermost membranes of primary and secondary plastids [35]. The ClpB/HSP100 is thought to associate with TIC machinery and function in protein folding [36]. It is therefore possible that *Tg*ClpB3/4/5 may be involved with the import of apicoplast proteins. Phenotype score of *Tg*ClpB3 is -4.75 based on a previous study that a genome-scale screen measures the contribution of each *T. gondii*’s gene [37]. Additionally, the failure to construct a viable *Tg*ClpB3-deficient parasite suggests an important function for this protein.

This study represents the first complete analysis of Clp chaperones and proteases in *T. gondii*. We showed that *Tg*ClpB1 and *Tg*ClpB2 are respectively present in parasitic cytoplasm and the mitochondria of *T. gondii*. Although the knockouts of *Tg*ClpB1/2 have no observed effect on the growth and replication of parasites at normal temperature, the loss of *Tg*ClpB1 resulted in a reduction of *T. gondii* replication and invasion rates after treatment at high temperature. *Tg*ClpB1 therefore plays a crucial role for the thermotolerant survival and growth of *T. gondii* tachyzoites. We also discovered a novel apicoplast-localized ClpB, *Tg*ClpB3, which is necessary for parasite viability.

## MATERIALS AND METHODS

### Ethics statement

Laboratory animal care and experimentation were performed in accordance with the Guide for the Care and Use of Laboratory Animals of the Ministry of Science and Technology of the People’s Republic of China. All protocols used for animal studies were approved by the Committee on the Ethics of Animal Experiments of the Harbin Veterinary Research Institute (HVRI) of the Chinese Academy of Agricultural Sciences (CAAS) (approval number BRDW-XBS-12).

### Cultivation of host cells and transfection of *T. gondii* tachyzoites

Human foreskin fibroblasts (HFFs) and Vero cells were maintained in DMEM supplemented with 10% heat-inactivated fetal bovine serum (FBS) (Gibco-BRL), 2 mM glutamine, and 0.5% penicillin-streptomycin. All mammalian cells were cultured at 37°C and 5% CO_2_ in tissue culture plates. Tachyzoites of *T. gondii* RH ΔHXGPRT- or RH, as well as derived transgenic strains generated in this study, were propagated in Vero cells under standard procedures. Heat shock experiments for extracellular parasites were performed by incubating at various temperatures prior to harvest.

To generate *Tg*ClpB1/2/3 knockout parasites, pCD-*Tg*ClpB1/2/3 plasmids were transfected into 1×10^7^ parasites by electroporation and selected by 1 μM pyrimethamine. To generate *Tg*ClpB1/2 complementing parasites, pBH-syno*Tg*ClpB1/2 plasmids were transfected into 1×10^7^ Δ*Tg*ClpB1/2 and selected by mycophenolic acid (20 μg/ml) and xanthine (50 μg/ml). Stable clones were isolated by limiting dilution in 96-well plates. Single clones were amplified and screened by PCR and Western blotting. Transgenic parasites overexpressing *Tg*ClpB1/2 were performed by electroporation of pBH-syno*Tg*ClpBs into 10^7^ HX tachyzoites. The pBS-DHFR-*Tg*PRX3-HA and pBS-DHFR-*Tg*ACP-HA plasmids were transfected into *Tg*ClpB2 and *Tg*ClpB3 respectively transgenic parasites and selected by 1 μM pyrimethamine.

### Bioinformatic procedures

Protein sequences from *Plasmodium falciparum* ClpP (accession no. PFC0310c), *Pf*ClpR (PF14_0348), *Pf*ClpB1 (PF08_0063), *Pf*ClpC (PF14_0063), *Pf*ClpQ (XP_001350699), *Pf*ClpY (PF3D7_0907400), *Escherichia coli* ClpP (P0A6G7), *Ec*ClpA (M31045), *Synechococcus elongatus* ClpX (PCC7942) and *Arabidopsis thaliana* ClpS1 (NP_564937) were retrieved from the GenBank and used in BLASTp queries of the ToxoDB (http://ToxoDB.org). Conserved regions in the catalytic domains of the Clp superfamily were analyzed by the NCBI conserved domain search program.

### Cloning procedures and plasmids construction

All the PCR amplifications were performed with either Ex Taq (TaKaRa) or the KOD-Plus-Neo (TOYOBO) kit. Primers are listed in Supplementary Table 1. The partial *Tg*ClpB1, *Tg*ClpB2 and *Tg*ClpB3 were individually cloned into pET-28a. Construction of the target gRNA expression constructs (*Tg*ClpB1/2; Figure 2) was performed by hierarchical fusion PCR assembly of the ToxoU6 promoter. *Tg*ClpB1, *Tg*ClpB2 and *Tg*ClpB3 target sequences along with the gRNA scaffold were then cloned into the PmeI site of pCD-Cas9 using the ClonExpress II Kit [38, 39]. The plasmids containing *Tg*ClpB1, *Tg*ClpB2 and *Tg*ClpB3 targeting fragments, respectively designated pCD-*Tg*ClpB1/2/3, were then used to establish knockout strains stably expressing GFP. The full ORFs of *Tg*ClpB1-FLAG, *Tg*ClpB2-FLAG, *Tg*ClpB3-FLAG, *Tg*GRA16-HA and *Tg*GRA24-HA were amplified and inserted into the EcoRV site of the pBS-HXGPRT vector under control of the TgGRA1 promotor. *Tg*PRX3 and *Tg*ACP open reading frames with a C-terminal HA tag were inserted downstream of the *Tg*GRA1 promoter, and a dihydrofolate reductase (DHFR) cassette was inserted upstream, to generate pBS-DHFR-*Tg*PRX3-HA and pBS-DHFR-*Tg*ACP-HA.

### Production of *Tg*Clp1, *Tg*Clp2 and *Tg*Clp3 mouse antisera

RNA was isolated from a RH strain of *T. gondii*, and cDNAs were created using the SuperScript III First Strand Kit (Invitrogen). The truncated *Tg*ClpB1 (amino acids 82-260, *TG*GT1_268650), *Tg*ClpB2 (amino acids 65-312, *TG*GT1_268650) and *Tg*ClpB3 (amino acids 472-654, *TG*GT1_275690) were each amplified by PCR, cloned into the pET28 vector, and used to transform BL21 (DE3) competent *E. coli*. Recombinant *Tg*ClpB1, *Tg*ClpB2 and *Tg*ClpB3 were purified by a Ni-NTA affinity chromatography according to the manufacturer’s instructions (Amersham Pharmacia Biotech, USA). The purified r*Tg*ClpB1, r*Tg*ClpB2 and r*Tg*ClpB3 emulsified with Freund’s complete adjuvant (Sigma Chemicals, USA) and immunized into BALB/c mice intraperitoneally. Three months after the immunization, antisera were collected and probed using Western blotting and an indirect fluorescent-antibody test (IFAT). The anti-*Tg*SAG2, anti-*Tg*tubulin, anti-*Tg*GRA7 and anti-*Tg*TCTP (*TG*GT1_251680) obtained by rabbit and mouse immunization.

### Immunofluorescence assay and confocal microscopy

HFF cells infected with *T. gondii* were fixed with 4% paraformaldehyde for 30 min and then permeablized with 0.03% Triton X-100 in PBS at 4°C for 10 min. The cells were washed in PBS three times and blocked with 3% bovine serum albumin (BSA) at room temperature for 1 h. *Tg*ClpB1, *Tg*ClpB2 and *Tg*ClpB3 were detected with mouse polyclonal anti-FLAG M2 primary antibody and secondary antibodies conjugated to Alexa Fluor 488 fluorescent (life technology). *Tg*SAG2 protein was detected by rabbit polyclonal anti-*Tg*SAG2 and Alexa Fluor 647 conjugated goat anti-rabbit IgG. *Tg*PRX3-HA as *T. gondii* mitochondria marker and *Tg*ACP-HA as an apicoplast marker. Both were detected with mouse anti-HA primary antibody and a secondary antibody conjugated to Alexa Fluor 647 fluorescent (life technology), respectively. Nuclei were visualized with 4’, 6-diamidino-2-phenylindole (DAPI) stain. Cells were mounted with ProLong Diamond Antifade Mountant (Thermo Fisher Scientific). The samples were imaged using confocal laser-scanning microscopy (Leica).

### Improved mitochondrial targeting sequence

The fusion protein with HA tag was generated by linking the first 113 amino acids of to the full open reading frame of luciferase. The *Tg*ClpB2 transit sequence was amplified from tachyzoite *Tg*ClpB2 containing the full open reading frame by using sense primer *Tg*Lucb-DHFR-1F and antisense primer *Tg*Lucb-DHFR-1R for *Tg*ClpB2_113_-Luc-HA plasmid. *T. gondii* RH-WT were transfected and selected by pyrimethamine. Immunolabeling of *Tg*ClpB2_113_-Luc-HA fusion proteins was performed using the anti-HA monoclonal antibody and anti-*Tg*SAG2 antibody. Fluoresce was observed by a confocal microscope.

### Western blot analyses

Extracellular free parasites were harvested by centrifuging at 3000 g for 10 min and then lysed by RIPA (Sigma) for 10 min in ice. The samples were loaded on 10% SDS-PAGE and transferred at 60 mA for 150 min to the PVDF membrane Immobilon-PSQ (Millipore). The membrane was blocked for 60 min at room temperature with 3% skim milk in PBS. The primary antibodies were diluted as follows: mouse anti-*Tg*ClpB1 (1:500), mouse anti-*Tg*ClpB2, mouse anti-*Tg*ClpB3, rabbit anti-*Tg*Tubulin (1:2000), mouse anti-*Tg*TCTP (1:500) and mouse anti-FLAG (1:2000) (Sigma). A horseradish peroxidase-conjugated goat anti-mouse antibody (1:4000) was used as the secondary antibody. The immunoblots were visualized on blue-sensitive x-ray film using the SuperSignal West Pico Chemiluminescent Substrate kit (Thermo Fisher Scientific).

### Plaque assay

HFF monolayers grown in 6-well plates were infected with 100 parasites per well. After incubation for 3 h, wells were washed with PBS and fresh media was added. The parasites were grown for 1 week before being fixed with cold methanol for 30 min. Documentation was performed with EVOS FL Auto Cell Imaging System at 40-fold magnification. Images were further processed with Photoshop.

### Intracellular replication assay

To determine the replication efficiency, the freshly egressed parasites were plated onto HFF cells in 6-well plates and allowed to invade for 3 h. After washing with PBS three times, parasites were allowed to grow for 24 h. IFA was performed using the anti-*Tg*SAG2 antibody (1:1000). The number of parasites per vacuole was determined in 100 vacuoles for three independent experiments.

### Red/green invasion assays

The 5×10^5^ freshly egressed parasites were added to confluent HFF cells in 12-well plates. Parasites were allowed to invade for 2 h and were then fixed with 4% paraformaldehyde. After blocking with 3% BSA in PBS, The attached parasites were labeled with rabbit polyclonal anti-*Tg*SAG2 antibody followed by Alexa Fluor 594 conjugated goat anti-rabbit IgG without permeabilization. Furthermore, the parasite infected cells were permeabilized with 0.03% Triton X-100 in PBS at 4°C for 10 min. Released parasites were labeled with rabbit polyclonal anti-*Tg*SAG2 followed by Alexa Fluor 488 conjugated goat anti-rabbit IgG. The number of extracellular and intracellular parasites counted in 100 parasites for three independent experiments.

### Induced egress assay

Freshly released parasites were allowed to invade the confluent HFF monolayers grown on 6 well plates for 36 h. The parasite infected HFF were artificially inducing egress with prewarmed egress media containing 0.06% DMSO or 1 μM A23187 (Sigma) calcium ionophore for 10min at 37°C in the dark. Parasites were fixed with cold methanol for 30min and were stained with anti-*Tg*GRA7 and anti-*Tg*SAG2 (1:1000) antibodies after blocking with 2% BSA in PBS. The average number of egressed vacuoles was determined by counting 100 vacuoles for three independent experiments for each condition.

### Statistical analysis

The graphs and All statistical analyses used in the work were performed by GraphPad Prism version 5.00 (GraphPad Software, San Diego, CA). The various assay conditions used herein were evaluated with a Student’s t-test and one-way analysis of variance (ANOVA) followed by Tukey’s multiple comparison test with a 95% confidence interval. Values of P < 0.05 were considered statistically significant.

## SUPPLEMENTARY MATERIALS FIGURES AND TABLE


