# Supplementary material for: Toxoplasma gondii Clp family protein: TgClpB1 plays a crucial role in thermotolerance
Source: Oncotarget. 2017 Sep 18;8(49):86117–29. doi: 10.18632/oncotarget.20989 (PMC5689671; doi:10.18632/oncotarget.20989)
Supplement: Supplementary file 1 [file oncotarget-08-86117-s001.pdf]

Toxoplasma gondii Clp family protein: *Tg*ClpB1 plays a crucial role in thermotolerance

SUPPLEMENTARY MATERIALS

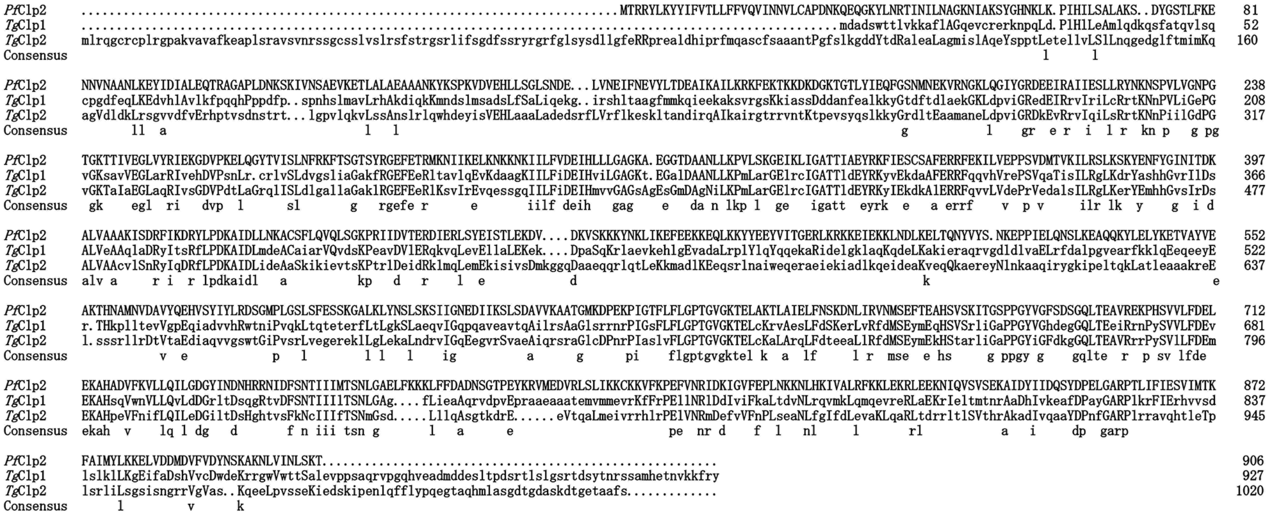

Supplementary Figure 1: Polypeptide alignments of the full open reading frame of PfClp2, TgClp and TgClp2.

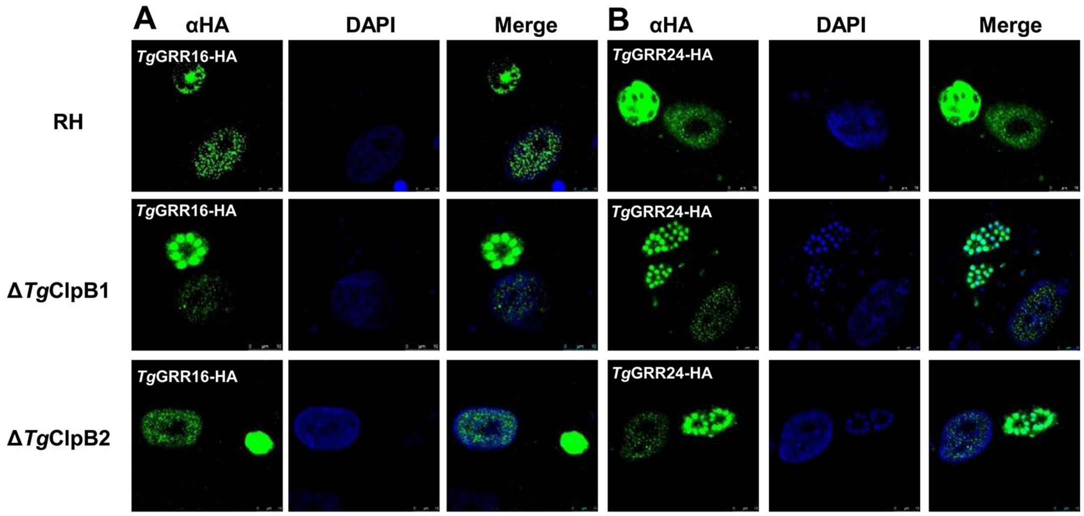

Supplementary Figure 2: Neither TgClpB1 nor TgClpB2 is not required for the translocation of TgGRA16 and TgGRA24 to the host nucleus. HFFs were infected with RH-WT, ΔTgClpB1 or ΔTgClpB2 strains expressing HA-tagged TgGRA16 **(A)** and TgGRA24 **(B)**, respectively. After 24 h, the cultures were washed, fixed, and stained with antibodies to the HA tag.

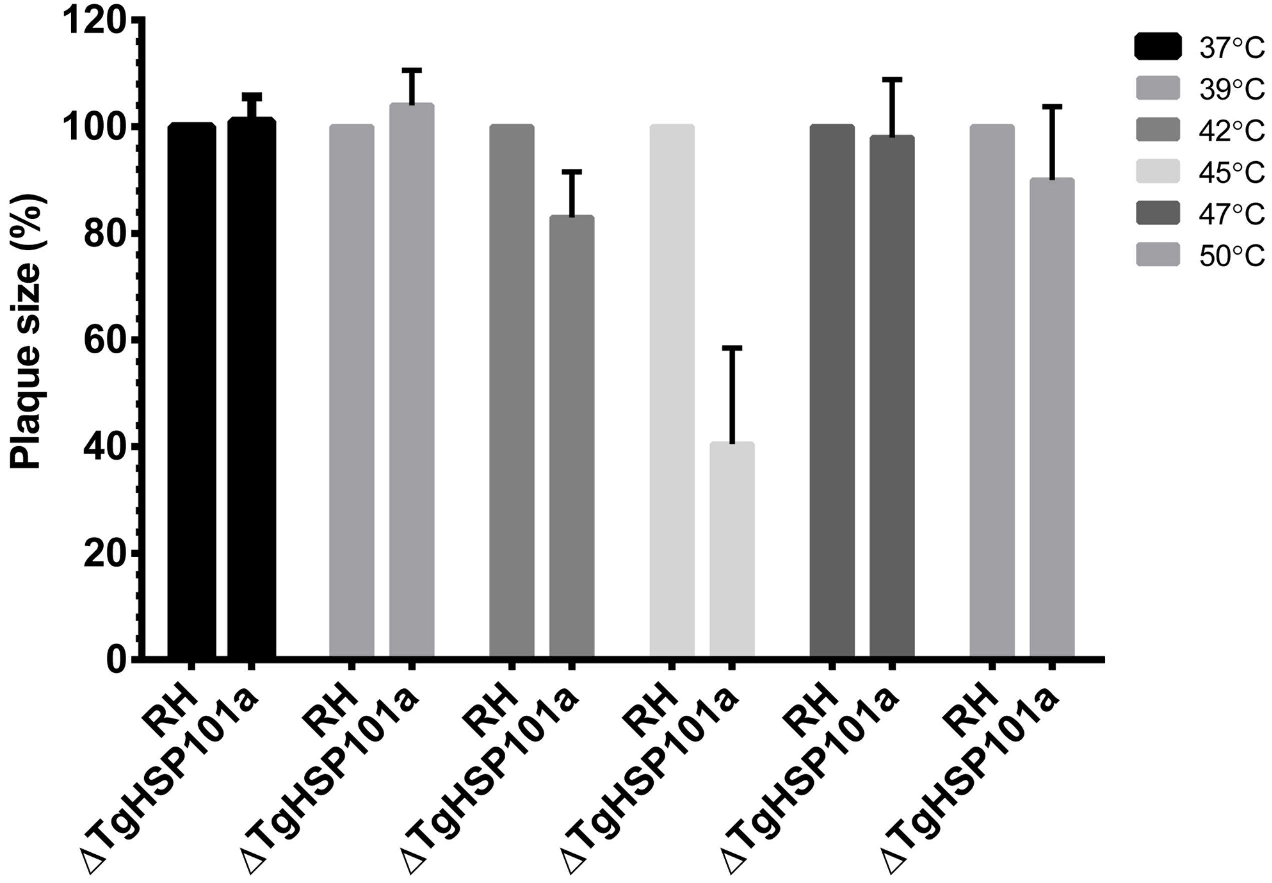

Supplementary Figure 3: Growth analysis of the indicated parasites grown on HFF cells for 7 days. When parasites were grown in HFF cells after the released parasites treated at 37, 39, 42, 45, 47, 50°C, plaque sizes of ΔTgClpB1 was determined and compared with RH-WT.

Supplementary Table 1: Primer sequences used to amplify and sequencing

Primer name	Sequence
TgClpB1_Ex_F	AATGGGTCGCGGATCCAGCCCGAACCACTCGCTCAT
TgClpB1_Ex_R	GGTGGTGGTGCTCGAGCACCGCAGTCAATCGCTCC
TgClpB2_Ex_F	AATGGGTCGCGGATCCGGTCGCTTTGGTCTGTCGT
TgClpB2_Ex_R	GGTGGTGGTGCTCGAGGATGATGGGATTGTTCTTA
TgClpB3_Ex_F	AATGGGTCGCGGATCCATTGAGAAGGACAAGGCACT
TgClpB3_Ex_R	GGTGGTGGTGCTCGAGTTCGGCAGCTCTGTTTAGG
TgHSPClpB1_gRNA_1S	CCACTAGTTCTAGAGCGGCCGTTTAAACGAGCTCCAAGTAAGCAGAAG
TgHSPClpB1_gRNA_1aS	CGGAGCAGGAAAGACCGAAGAACTTGACATCCCCATTTAC
TgHSPClpB1_gRNA_2S	CTTCGGTCTTTCCTGCTCCGGTTTTAGAGCTAGAAATAGC
TgHSPClpB1_gRNA_2aS	CCGTATTACCGCCTTTGAGTGTTTAAACGAGCTGATACCGCTCGCC
TgHSPClpB2_gRNA_1S	CCACTAGTTCTAGAGCGGCCGTTTAAACGAGCTCCAAGTAAGCAGAAG
TgHSPClpB2_gRNA_1aS	CGGTACCTACTGCTGAAATCAACTTGACATCCCCATTTAC
TgHSPClpB2_gRNA_2S	GATTTCAGCAGTAGGTACCGGTTTTAGAGCTAGAAATAGC
TgHSPClpB2_gRNA_2aS	CCGTATTACCGCCTTTGAGTGTTTAAACGAGCTGATACCGCTCGCC
TgHSPClpB3_gRNA_1S	CCACTAGTTCTAGAGCGGCCGTTTAAACGAGCTCCAAGTAAGCAGAAG
TgHSPClpB3_gRNA_1aS	GCGGGGAGTTAAGCGCCGACAACTTGACATCCCCATTTAC
TgHSPClpB3_gRNA_2S	GTCGGCGCTTAACTCCCCGCGTTTTAGAGCTAGAAATAGC
TgHSPClpB3_gRNA_2aS	CCGTATTACCGCCTTTGAGTGTTTAAACGAGCTGATACCGCTCGCC
TgClpB1_C_1F	CCGCTCTAGACTAGTGGATCCATGGACGCGGACTCCTGGACGAC
TgClpB1_C_1R	CTTTCCTGCACCGAGTATGACGTGTA
TgClpB1_C_2F	TACACGTCATACTCGGTGCAGGAAAG
TgClpB1_C_2R	GGTACCGGGCCCCCCCTCGAGTCAGTATCTGAATTTCTTGACATTC
TgClpB2_C_1F	CCGCTCTAGACTAGTGGATCCATGCTGCGCCAGGGTTGT
TgClpB2_C_1R	GACAGACCAAAGCGACCTCTATATCGTGAGCTGAAATCC
TgClpB2_C_2F	GGATTTCAGCTCACGATATAGAGGTCGCTTTGGTCTGTC
TgClpB2_C_2R	GGTACCGGGCCCCCCCTCGAGTCACTTATCGTCGTCATCCTTGTAATCCGAGAACGCAGCCGTTTCC
TgGRA16_HX_F	AAGAAGCTTGATGGGGATATCATGTATCGAAACCACTCAGG
TgGRA16_HX_R	GTCGTACGGATACATGATATCTCAAGCGTAGTCTGGGACGTCGTATGGGTACATCTGATCATTTTTCC
TgGRA24_HX_F	AAGAAGCTTGATGGGGATATCATGCTCCAGATGGCACGATATAC
TgGRA24_HX_R	GTCGTACGGATACATGATATCTTAAGCGTAGTCTGGGACGTCGTATGGGTAATTACCCTTAGTGGGTGGTTTAAC
TgPRX3_DHFR_F	AAGAAGCTTGATGGGGATATCATGGCGGCTTGCCTTCGAGCGGCCCGTTTGTCTCTCAGACAAATGGAAGGCTTGATA
TgPRX3_DHFR_R	GTCGTACGGATACATGATATCTTAAGCATAATCTGGAACATCATATGGATAGTTTTTCAGTTGTCCAAGGTAG
TgHSPClpB1_SEQ_F	ATCGCCGGCGCCAAGTTCC
TgHSPClpB1_SEQ_R	GGTAAAGCGCCTCATCTC
TgHSPClpB2_SEQ_F1	AGGATGCTCCTCCTTAGTTT
TgHSPClpB2_SEQ_R1	GCACGAGAAGTTCGGTTT
TgHSPClpB2_SEQ_F2	AGCCCGAACTGGTAATGC
TgHSPClpB2_SEQ_R2	AGTGCGGCGACTGAGGAT

(Continued)

Primer name	Sequence
TgGRA16_SEQ_F	AACCACTCAGGGATACGGC
TgGRA16_SEQ_R	CGGACTTCGCTTTGAACACTT
TgCAS9_SEQ_1S	CCACTAGTTCTAGAGCGGCCGTTTAAACGAGCTCCAAGTAAGCAGAAG
TgCAS9_SEQ_2AS	CCGTATTACCGCCTTTGAGTGTTTAAACGAGCTGATACCGCTCGCC
TgLucb-DHFR-1F	AAGAAGCTTGATGGGGATATCATGCTGCGCCAGGGTTGT
TgLucb-DHFR-1R	GTTTTTGGCGTCTTCCATGTAGTCATCTCCTTTCAGAG
TgLucb-DHFR-2F	CTCTGAAAGGAGATGACTACATGGAAGACGCCAAAAAC
TgLucb-DHFR-2R	GTCGTACGGATACATGATATCTTAAGCATAATCTGGAACATCATATGGATACAATTTGGACTTTCCG
